# MHC-I Molecules Selectively Inhibit Cell-Mediated Cytotoxicity Triggered by ITAM-Coupled Activating Receptors and 2B4

**DOI:** 10.1371/journal.pone.0107054

**Published:** 2014-09-16

**Authors:** Rubén Corral-San Miguel, Trinidad Hernández-Caselles, Antonio José Ruiz Alcaraz, María Martínez-Esparza, Pilar García-Peñarrubia

**Affiliations:** Department of Biochemistry and Molecular Biology B and Immunology, School of Medicine, University of Murcia, Campus of International Excellence “Campus Mare Nostrum” and IMIB (Instituto Murciano de Investigaciones Biosanitarias)-Arrixaca, Murcia, Spain; Centre de Recherche Public de la Santé (CRP-Santé), Luxembourg

## Abstract

NK cell effector functions are controlled by a combination of inhibitory receptors, which modulate NK cell activation initiated by stimulatory receptors. Most of the canonical NK cell inhibitory receptors recognize allelic forms of classical and non-classical MHC class I molecules. Furthermore, high expression of MHC-I molecules on effector immune cells is also associated with reverse signaling, giving rise to several immune-regulatory functions. Consequently, the inhibitory function of MHC class I expressed on a human NKL cell line and activated primary NK and T cells on different activating receptors are analyzed in this paper. Our results reveal that MHC-I molecules display specific patterns of “selective” inhibition over cytotoxicity and cytokine production induced by ITAM-dependent receptors and 2B4, but not on NKG2D. This contrasts with the best known “canonical” inhibitory receptors, which constitutively inhibit both functions, regardless of the activating receptor involved. Our results support the existence of a new fine-tuner inhibitory function for MHC-I molecules expressed on cytotoxic effector cells that could be involved in establishing self-tolerance in mature activated NK cells, and could also be important in tumor and infected cell recognition.

## Introduction

The mechanisms that control the activity of NK and other cytotoxic effector cells are determined by a fine balance between signals triggered by activating and inhibitory receptors, which ultimately determine the activation of the effector cell [1]–[2]. Regarding cytotoxicity, several NK cell-activating receptors may directly recognize ligands expressed on the surface of infected or stressed tumor target cells [1]–[2]. In addition to cytolytic activity, NK cells produce immunoregulatory cytokines such as IFN-γ, TGF-β, IL-1, IL-10, GM-CSF and chemokines when triggered by activating receptors [1]–[2]. The role of inhibitory receptors in this human NK cell immunoregulatory function has not been totally established. Inhibitory receptors antagonize NK cell responses through the recruitment of the protein tyrosine phosphatases, SHP-1 and SHP-2, to their ITIM (Immunoreceptor Tyrosine-based Inhibitory Motif) sequences [1]–[2]. Despite the complexity of the target recognition process, NK cells maintain self-tolerance, a function that is also achieved by a combination of inhibitory receptors that modulate the NK cell activation process initiated by activating receptors [3]–[4]. The best studied human (canonical) NK cell inhibitory receptors, Killer Ig-like receptors, (KIRs), Leukocyte Ig-like receptors (LILRs) and lectins-like receptors such as CD94/NKG2A, mediate self-tolerance through chronic cognate interaction with their ligands, mainly MHC (Major Histocompatibility Complex) class I molecules expressed on target cells. Thus, loss of MHC-I expression by virus-infected or tumor cells leads to NK cell activation as proposed by the “missing-self hypothesis” [1]–[3]. Additionally, it seems that the MHC-I environment redesigns NK cell receptor expression and reactivity [4]. Hence, mouse NK cells that express inhibitory receptors specific for self-MHC are more responsive than their non-expressing counterparts [5]. On the other hand, MHC-I-deficient mice display reduced responsiveness despite having self–tolerant NK cells [6].

Beside their classical function concerning antigen presentation and self-tolerance, MHC class I molecules can also mediate reverse signaling after aggregation, and display non-classical functions [7]–[9]. In this respect, previous studies from our laboratory have shown that crosslinking MHC-I on the membrane of human cytolytic effector cells induces intracellular tyrosine phosphorylation and inhibits the cytotoxicity directed against tumor cells [10]–[12]. Furthermore, constitutively expressed MHC class I molecules on macrophages protect mice from sepsis by attenuating TLR-triggered inflammatory responses [13]. These findings demonstrate that MHC class I molecules can act not only as ligands, but also as signaling receptors able to mediate reverse signaling through direct aggregation or association with other receptors.

This work further explores the role of MHC-I molecules expressed on human activated NK and T cells triggered by different activating receptors. The results show that MHC class I proteins exert an inhibitory function on both NK cell-mediated cytotoxicity and IFN-γ production, depending on the particular killer activating receptor triggered in the activated effector cells. Therefore, besides the well known role of MHC-I molecules expressed on target cells, NK cell upregulation of MHC class I could constitute a novel mechanism of immune-regulation, tolerance and evasion of tumor or infected cells.

## Materials and Methods

### Antibodies

The anti-HLA class I mAb used were: W6/32 (IgG2a, obtained from ATCC), BB7.7 (IgG2b, which recognizes a combinatorial determinant of HLA-A, B and C and β_2_- microglobulin; obtained from ATCC), KD1 (IgG2a, anti-CD16), HP-3B1 (IgG2a, anti-CD94), Z199 (IgG2b, anti-heterodimer CD94/NKG2A) and HP-F1 (IgG1, anti-ILT2) were kindly provided by Dr. A. Moretta (Milan, Italy) and Dr. M. López-Botet (Barcelona, Spain). All of them were supernatants from hybridoma cultures. 3D12 (IgG1, anti-HLA-E obtained from eBioScience, San Diego, CA). Anti-NKG2D (clone 1D11, IgG1) was from eBioscience, anti-NKp46 (IgG2b) was from RD Systems and anti-2B4 (C1.7, IgG1) was from Immunotech. Isotype control Abs were from Sigma-Aldrich. HLA-G membrane expression was assessed with an MEM-G9 mAb specific for HLA-G H chain associated with β_2_-microglobulin, and MEM-G1 mAb, specific for the HLA-G free H chain molecule were from Exbio (Praha, CZ). The conjugated mAb FITC-anti-CD3, FITC-anti-CD25, PE-anti-CD56 and PE- and Cy5-anti-CD16 were from Becton Dickinson.

### Effector and target cells

The human NK cell line NKL (kindly provided by Dr Michael J. Robertson, Indiana University (Bloomington, IN)) [14] was cultured at a growth rate ranging from plateau to exponential phase (approx. from 0.05×10^6^ cells/ml to 0.6×10^6^ cells/ml) for up to 24–48 h, using complete tissue culture medium (RPMI 1640, 10% heat-inactivated FBS and antibiotics; Gibco) supplemented with 100 U/ml rIL-2 (Proleukin; Chiron). Additionally, to increase NKL killer activity, cells were grown in the presence of 1000 U/ml rIL-2 for 24–48 h.

Polyclonal primary NK cells were obtained from the blood of healthy volunteers. Protocols were approved by the Ethics Committee of the University of Murcia and complied with the Helsinki Declaration and the Good Clinical Practice guidelines. Volunteers always gave written informed consent. Peripheral blood lymphocytes (PBL) were stimulated with irradiated allogeneic cells as previously described [15], in the presence of IL-15 (25 ng/ml) (R&D systems). After stimulation, activated cells were maintained in IL-2-supplemented (100 U/ml) TCM until cellular quiescence (low expression of CD25). NK cells were then purified by negative selection using anti-CD3 (OKT3) and goat anti-mouse-coated magnetic beads (Dynabeads, Invitrogen). The resulting populations were shown to be >95% CD3^−^CD16^+^CD56^+^ by flow cytometry.

Target cells used were mouse mastocytoma FcR^+^ P815 (ATCC) grown in TCM.

### Immunofluorescence analysis

Phenotypic analysis of cells was carried out by direct or indirect immunofluorescence staining, depending on whether or not the primary mAb was fluorochrome-conjugated or unconjugated, respectively, on a FACScan cytometer (Becton-Dickinson) as previously described [10]. Cytofluorometer data were analysed using the CellQuest program (Becton-Dickinson). A minimum of 4000 events per sample was analyzed.

### Cytotoxicity assays

The cytotoxic activity of human NK cells was tested in 4 h ^51^Cr release assays [10]. Target cells were labeled with Na^51^_2_CrO_4_ (100 µCi^−^[3.7 MBq]/10^6^ cells; PerkinElmer) for 1.5 hour at 37°C. Target cells were washed and seeded on U-bottom 96-well plates at 5000 cells per well. Effector cells were then added in 100 µL medium at different E/T (Effector/Target) ratios.

The redirected lysis assays were performed using ^51^Cr-labeled P815 target cells pre-incubated with optimized concentrations of the following mAb against activating and/or inhibitory receptors or control Ig: anti-CD16 (culture supernatant), anti-NKp46 (0.34 µg/ml), NKG2D (0.34 µg/ml), 2B4 (0.17 µg/ml), anti-MHC-I (culture supernatant), anti-ILT2, anti-NKG2A or anti-CD94 (culture supernatants) and isotype control Ig (1.25 µg/ml). Effector cells were added at different E/T ratios and incubated for 4 h at 37°C. Then, supernatants were collected to determine ^51^Cr release, and the percentage of lysis was calculated as follows:



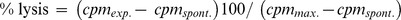



The cpm_spont_ was <15% of cpm_max_ in all experiments.

### Cytokine production assays

For cytokine production studies, NKL (25000 cells/well, grown in 100 U/ml rIL-2) or purified polyclonal NK cells (50000 cells/well) were co-cultured with P815 cells at a 1∶1 E/T ratio in the presence of the same combination of mAb, using twice the Ab concentration used for the cytotoxicity assay. After 22–24 h incubation, cell free supernatants were collected and tested for IFN-γ concentration by ELISA (eBioscience).

### Calculating the percentages of inhibition of cytotoxicity and cytokine production

The results, reported as the percentage of inhibition, were calculated as follows:



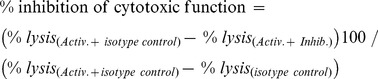





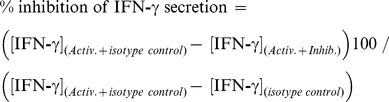



### Statistical analysis

Data are reported as mean ±SD. Statistical differences were analyzed using the Mann-Whitney U test, and p values lower than 0.05 were considered to indicate statistical significance. Calculations were performed using the SPSS 21.0 software (Chicago, IL, USA).

## Results

### The co-ligation of MHC-I expressed on NKL cells with different activating receptors selectively inhibits their cytolytic activity

**Figure 1:**
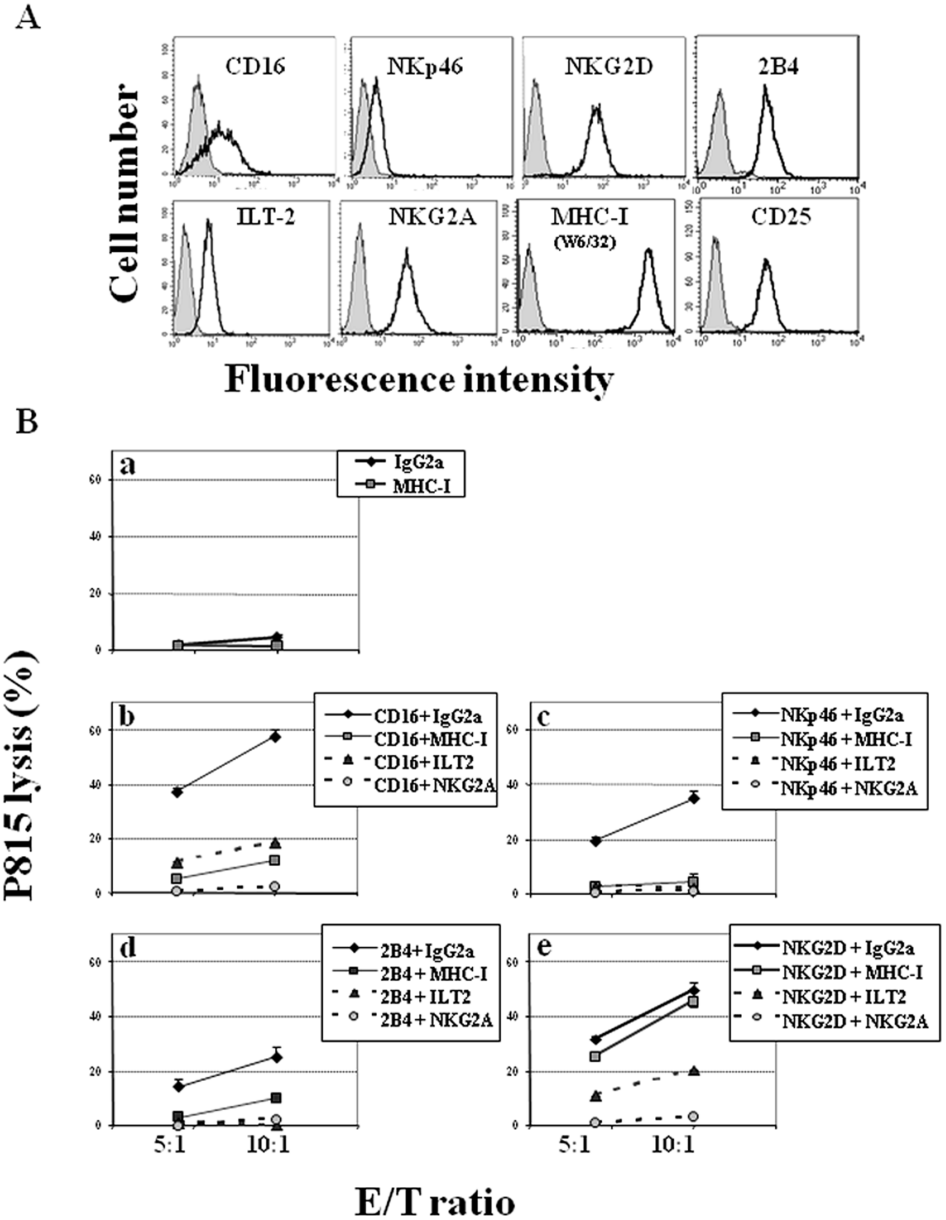
MHC-I engagement selectively inhibits cytotoxicity on NKL cells. (**A**) Exponentially growing NKL cells (see *Material and Methods* section) were phenotyped by flow cytometry. Filled histograms represent isotype control and open histograms represent surface receptor stained cells. (**B**) NKL cells were co-cultured with ^51^Cr-P815 cells in the presence of mAb: IgG2a isotype control or anti-MHC-I (**a**), against KAR (CD16 (**b**), NKp46 (**c**), 2B4 (**d**), and NKG2D (**e**)), plus control IgG2a or anti-MHC-I, anti-ILT2 or anti-NKG2A inhibitory receptors. The figure depicts one representative assay out of three performed with similar results.

We have previously shown that MHC-I can transduce inhibitory signals upon engagement with putative ligands expressed on the surface of K562 target cells [10], [12]. Here, we study the inhibitory effect of NK cell-expressed MHC-I molecules on the cytolytic activity triggered by different Killer Activating Receptors (KARs). To accomplish this, we use the redirected cytotoxicity assay against the FcR^+^ P815 murine cell line. To validate this assay, we first determined the basal level of P815 target cells killing by NKL (3.6±2.7% lysis at 20∶1 E/T ratio). The low level observed indicated that any interaction of NKL cell-activating receptors with putative murine ligand(s) was not significant in this experimental setting. Next, taking advantage of the fact that two different receptors may be co-ligated on the membrane of effector cells, NKL cells were triggered with optimal concentrations of mAb against CD16, NKp46 (CD335), NKG2D (CD314) or 2B4 (CD244) KARs, together with one of the following: isotype control Ab, anti-MHC-I (W6/32) or mAb specific for the canonical inhibitory receptors ILT2 or CD94/NKG2A (as positive controls of inhibition). Constitutive NKL cell expression of these cell markers is shown in **Figure 1A** **.** As negative control we previously determined that co-ligation of CD16, NKG2D and NKp46 with other NKL cell membrane receptors, such as CD2, CD58, CD54, CD50, CD29, CD44 and CD25, had no significant effect in this assay compared with mAb W6/32 (Figure S1 in File S1). **Figure 1B** **(panel a)** shows that anti-MHC-I, or its isotype-matched mAb alone, did not affect NKL-mediated P815 killing. However, anti-MHC-I mAb W6/32 was able to strongly reduce the killing of P815 triggered by anti-CD16 (91.3±8.3% inhibition, **Fig. 1B** **, panel b**), anti-NKp46 (90.8±5.3% inhibition, **Fig. 1B** **, panel c**), at 5∶1 and 10∶1 E/T ratio, or anti-2B4 mAb (70.3±18.0% inhibition **Fig. 1B** **, panel d**) at 5∶1 and 10∶1 E/T ratio. In contrast, the same concentration of anti-MHC-I mAb could not significantly reduce the killing triggered by NKG2D (**Fig. 1B** **, panel e**), which produced only 12.6±15.9% inhibition, when the E/T ratio was 5∶1 and 10∶1. As expected, anti-ILT2 or anti-CD94/NKG2A mAb completely neutralized the lysis of P815 induced by every single activating mAb used (**Fig. 1B** **, panels b–e**). Altogether, these results confirm that MHC-I molecules play an inhibitory role in the membrane of NKL cells.

To discard the possibility of an artefactual inhibition due to competition between activating and inhibitory mAb for P815 FcR, the percentages of NKL/P815 conjugation under the experimental conditions described above were determined. The results shown in Figure S2 in File S1 supported that the selective inhibition exerted by MHC-I molecules over CD16-, NKG2D- and NKp46-mediated NKL cytotoxicity in the reverse assays is not caused by a FcR competition phenomenon.

### Co-engagement of MHC-I with different activating receptors selectively inhibits the cytolytic activity of activated primary human NK and T cells

**Figure 2:**
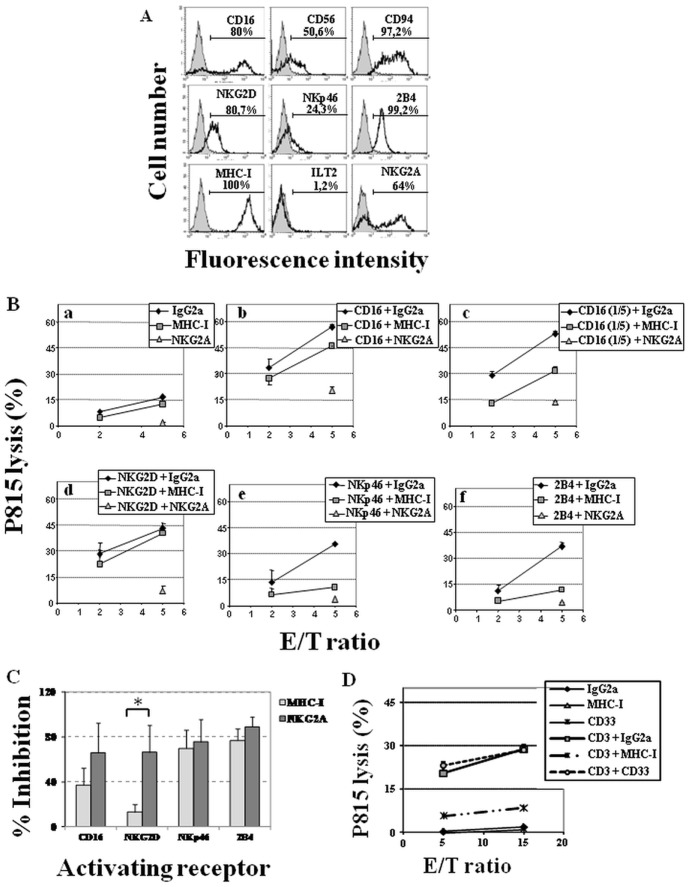
MHC-I engagement selectively inhibits cytotoxicity on activated human primary NK cells triggered by CD16, NKp46 or 2B4 but not by NKG2D activating receptors. (**A**) Phenotype of activated but quiescent polyclonal NK cells. Filled histograms represent isotype control and open histograms represent surface receptor stained cells. Numbers are percentages of positive cells in each panel. Data show one representative donor out of six tested in this study. (**B**) Purified quiescent NK cells were co-cultured with ^51^Cr-P815 cells at 2∶1 and 5∶1 E/T ratios in the presence of mAb IgG2a isotype control, anti-MHC-I or anti-NKG2A (**a**), or against KAR (CD16 undiluted (**b**), CD16 diluted 1/5 (**c**), NKG2D (**d**), NKp46 (**e**) and 2B4 (**f**)), plus control Ig, anti-MHC-I or anti-NKG2A mAb. One representative donor (n = 6) is shown. (**C**) Inhibition percentages (mean ±SD) for each inhibitory receptor in all performed assays. Statistically significant difference comparing MHC-I *versus* NKG2A inhibitory effect is presented, *p = 0.034. (D) **MHC-I engagement selectively inhibits cytotoxicity on activated human T cells triggered by anti-CD3 activating receptor.** Purified activated T cells were co-cultured with P815 cells at 5∶1 and 15∶1 E/T ratios in the presence of mAb against CD3 plus control IgG2a, CD33 (WM53) or MHC-I (W6/32) mAb. One representative donor (n = 5) is shown.

Whether or not the inhibitory capacity of MHC class I molecules expressed on NKL cells is a general mechanism of inhibition in activated human NK cells was further studied. For this, polyclonal populations of activated NK cells from PBMC obtained from six healthy donors were expanded by co-culture with allogeneic cells in the presence of IL-15. Under these experimental conditions we were able to obtain a population of activated NK cells (from 40 to 85% of CD3^−^CD16^++^CD56^+^ cells among different donors) which were sub-cultured in IL-2 supplemented culture medium until quiescence (very low expression of CD25 antigen after 3–4 weeks of sub-cultures). NK cells were further enriched by negative selection, and then tested for the surface expression of every receptor included in the current study by flow cytometry analysis. **Figure 2A** shows the results obtained from a representative donor out of six performed with similar results. Most of these cells expressed CD16, CD56, CD94, NKG2D and 2B4 antigen (**Fig. 2A**) although, as has been described in freshly isolated PBLs [16], the expression of NKG2D varied among donors, with mean percentages of 67.2±23.5% from the six individuals tested. The percentages of NKp46^+^ cells also varied among individuals and were lower (34.2±24.5%) than those reported for resting human NK cells [17]. The percentages of ILT2^+^ cells were very low (6.7±5.7%), while NKG2A^+^ cells reached 48.1±11.9% in these NK cells (**Fig. 2A**). Furthermore, we found that these quiescent NK cells were mainly CD16^bright^CD56^dim^ and CD16^bright^CD56^−^ (**Fig. 2A**). Therefore, they could be phenotypically similar to the described CD56^dim^ subset of resting NK cells, which displays high natural cytotoxic ability after activation [18].

The results obtained in cytotoxicity assays were consistent with those obtained for NKL cells. Thus, MHC-I engagement could inhibit the cytotoxicity triggered by CD16 depending on the concentration of CD16 mAb assayed (**Fig. 2B** **panel b-c and 2C**). In agreement with results obtained with NKL, MHC-I could only slightly counteract (13.2±6.9% inhibition at E/T ratio of 5∶1) the effect of NKG2D-activating receptor (**Fig. 2B** **panel d and 2C**), whereas NKG2A effectively inhibited the specific P815 killing mediated by this activating receptor (66.3±24%). Furthermore, P815 lysis induced by NKp46 was highly decreased by co-ligation with MHC-I (69.9±16.2%), (**Fig. 2B** **panel e, and 2C**). Finally, 2B4 triggered cytotoxic activity was almost completely neutralized by NKG2A (88.9±8.9%), and anti-MHC-I was also able to notably decrease the killing effect (76.7±10.3%), (**Fig. 2B** **panel f and 2C)**. These results demonstrate and confirm that, contrarily to classic MHC-I recognizing inhibitory receptors, the inhibition of cytotoxicity after the engagement of MHC-I molecules on activated human NK cells is a selective phenomenon that depends on the specific activating receptor triggered.

To further study the inhibitory ability of MHC-I molecules on the activity triggered by ITAM-bearing activating receptors, we studied P815 redirected lysis by activated T cells from five donors, after co-ligation of MHC-I with CD3/TcR molecules (**Fig 2D**). Ab isotype and anti-CD33 mAb were used as negative control of inhibition since we found that mAb anti-CD33 is able to inhibit the cytotoxicity triggered by DAP10-coupled NKG2D, but not by receptors transducing through ITAM-bearing adaptors (manuscript submitted). As shown in primary NK cells, MHC-I engagement strongly reduced the CD3 triggered cytotoxicity (76.52±11.86 at E/T ratio of 5∶1) compared with the anti-CD33 mAb (WM53) (15.37±14.07) and the isotype control (0.28±0.46) at the same ratio. These results indicated that MHC-I molecules play an inhibitory role on ITAM-dependent cytotoxic activating signaling pathways.

### Co-engagement of MHC-I by others anti-MHC-I mAb with different NK activating receptors selectively inhibits cytolytic activity of NKL cells

**Figure 3:**
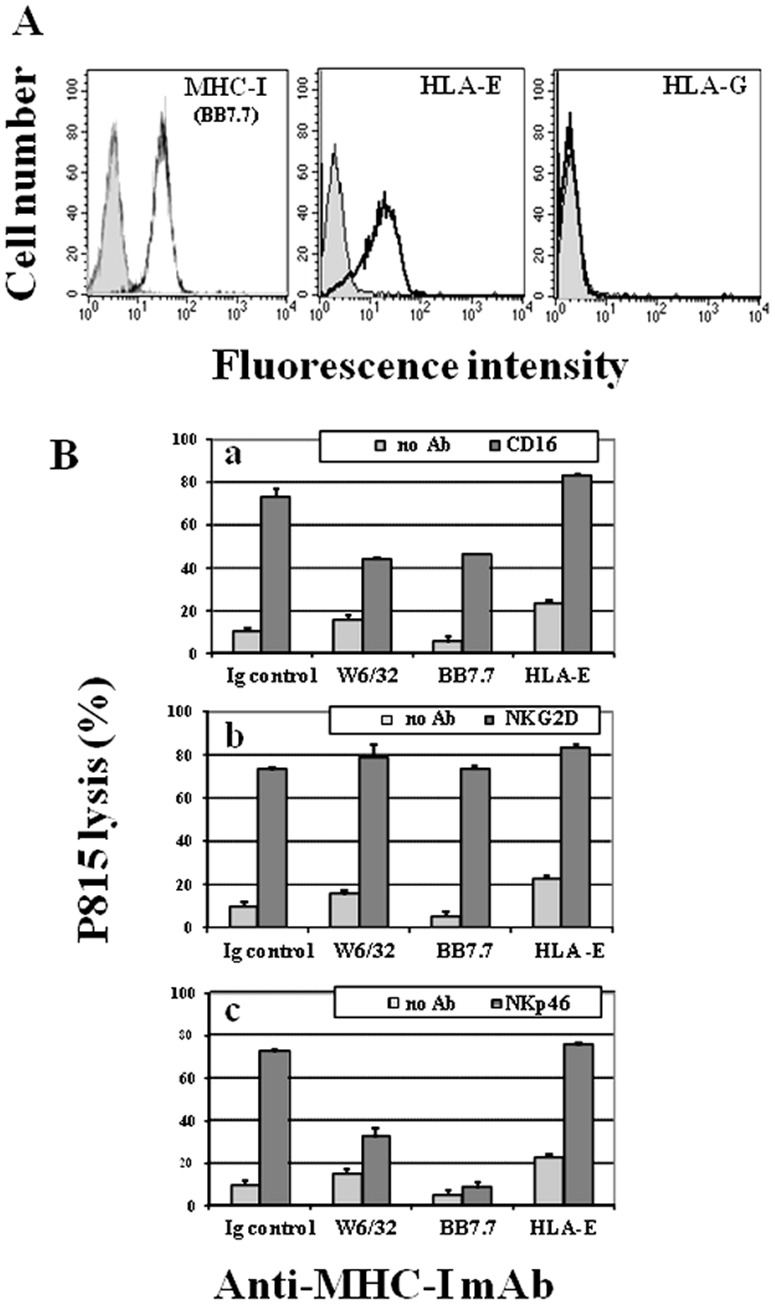
Classical MHC-I molecules are involved in selective killing inhibition of NKL cells triggered by CD16 and NKp46 activating receptors. (**A**) Classical and non-classical MHC-I expression on NKL cells. Filled histograms represent isotype control and open histograms represent surface receptor stained cells. (**B**) Exponentially growing NKL cells were co-cultured with ^51^Cr-P815 cells at 5∶1 E/T ratio in the presence of mAb against KAR (CD16 (**a**), NKG2D (**b**) or NKp46 (**c**)), plus control Ig, or different anti-MHC-I mAb (W6/32, BB7.7 or HLA-E clone 3D12). One representative experiment (n = 3) is shown.

Next we determined whether different MHC-I, classical and non-classical, molecules were expressed on NKL cells, and whether they exerted an inhibitory function in NK cell-mediated cytotoxicity. For this purpose, besides W6/32 (which recognizes the α3 domain of MHC-I) we used mAb BB7.7 (which recognizes a combinatorial determinant of the HLA-A, B and C and β2-microglobulin), the anti-HLA-E 3D12 mAb and anti-HLA-G mAb. Flow cytometry analyses revealed that the NKL cells were BB7.7^+^, HLA-E^+^ and HLA-G^−^ (**Figure 3A**). Redirected lysis experiments (**Figure 3B**) revealed that the mAb BB7.7 behaved similarly to W6/32, since both inhibited the cytotoxic activity mediated by CD16 and NKp46, although the inhibitory activity of BB7.7 on signals initiated by NKp46 was even stronger than that of W6/32 mAb. Consistent with the above results, none of them acted as inhibitor on cytotoxicity triggered by NKG2D. These results also suggest that the inhibitory function of MHC-I molecules involves the presence of β2-microglobulin and excludes the involvement of the HLA-E non-classical MHC-I protein.

### Inhibition of IFN-γ secretion by NK cells after co-ligation of MHC-I with different NK activating receptors

**Figure 4:**
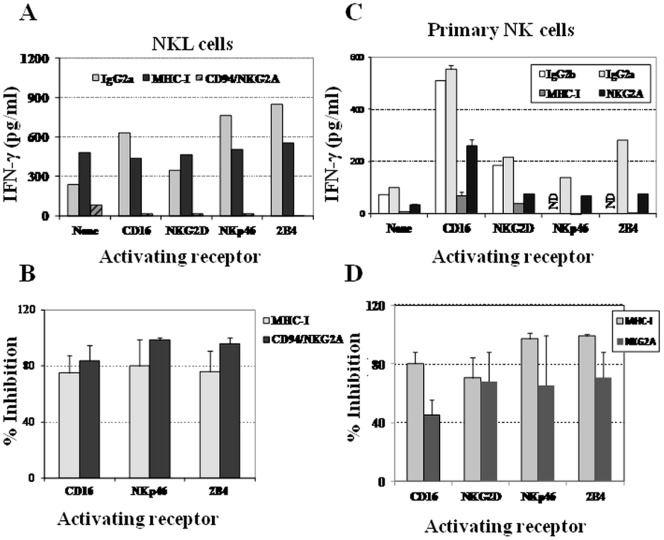
Inhibition of IFN-γ secretion by MHC-I in NKL and human activated NK cells. Exponentially growing NKL cells (**A and B**) were co-cultured with P815 cells at 1∶1 E/T ratio as described in *Materials and Methods*. (**A**) IFN-γ secretion is efficiently inhibited by anti-CD94 mAb in all cases. Anti-MHC-I mAb partially inhibits the secretion of IFN-γ induced by CD16, NKp46 and 2B4. Figure shows (**A**) one representative assay and (**B**) percentage of inhibition (mean ±SD) from the four experiments performed. (**C and D**) IFN-γ secretion by purified quiescent human activated primary NK cells is inhibited by anti-MHC-I mAb. Panel **C** shows one representative assay out of five (three different donors), and **D** the percentages of inhibition (mean ±SD) of anti-MHC-I and anti-NKG2A mAb. ND: not determined.

NK cells regulate cell–mediated immune responses by secreting a wide array of cytokines [1]–[2], [18]. Consequently, we next evaluated the secretion of IFN-γ by NKL cells co-cultured with P815 after co-ligation of either MHC-I or CD94/NKG2A with the activating receptors used above. First, it was checked that NKL cells did not produce IFN-γ when cultured alone or mixed with equal amounts of P815 cells (data not shown). **Figure 4A** displays the results obtained from one representative experiment out of four performed with similar results and **Figure 4B** shows the mean ±SD of percentages from four experiments. As expected, IFN-γ production was almost undetectable when cells were triggered by anti-CD94 mAb, unlike when IgG2a isotype control was used alone (**Fig. 4A**). In agreement with reported data [19] IFN-γ secretion was weakly induced by anti-NKG2D mAb in NKL cells, but it was secreted when cells were stimulated through CD16-, NKp46- or 2B4-activating receptors (**Fig. 4A**). Anti-CD94 mAb drastically inhibited the IFN-γ secretion induced by every activating receptor studied (from 94.0±11.1% to 98.7±1.6% of inhibition (**Fig. 4A and B**)). Regarding MHC-I molecules, W6/32 mAb was also able to partially inhibit the secretion of the IFN-γ induced by anti-CD16, anti-NKp46 or anti-2B4 mAb (75.1±12.5% to 80.1±19.0%), and occasionally induced a slight increase in IFN-γ production triggered by anti-NKG2D mAb (**Fig 4A-B**). In line with the results obtained with anti-CD94 mAb, anti-ILT2 mAb almost completely inhibited the IFN-γ production induced by all activating receptors studied on the NKL cell line (data not shown).

Next, we evaluated the ability of MHC-I to inhibit the secretion of IFN-γ in purified polyclonal activated NK cells. **Figure 4C** shows that anti-CD16 mAb was the best inducer of IFN-γ secretion as previously described for resting human NK cells [20]. The results indicated that, in this experimental setting, anti-MHC-I mAb almost completely inhibited the secretion of IFN-γ induced by every single activating receptor studied. In turn, anti-CD94/NKG2A mAb only partially inhibited the secretion of IFN-γ induced by the same activating receptors (**Figure 4C and D**). This diminished inhibition could be explained by the partial expression of NKG2A on these activated NK cell populations (**Figure 2A**).

Taken together, our results showed that, a) MHC-I molecules are selective inhibitors of both cytotoxicity and IFN-γ secretory function of NK cells, whereas b) canonical inhibitory receptors, such as CD94/NKG2A and ILT2, are able to prevent the secretion of this cytokine induced by most human activating receptors.

## Discussion

The present results further reinforce and extend experimental evidence from our laboratory concerning the inhibitory function triggered by MHC-I molecules expressed on NKL, human primary NK cells and a CD8^+^αβ T cell clone, K14B06 [10]–[12]. Herein we demonstrate that, similarly to the best known human inhibitory receptors, ILT2 and CD94/NKG2A, the inhibitory activity of MHC-I is strongly exerted on activating receptors, CD16 and NKp46, which transduce intracellular signals by association with ITAM-bearing adaptor molecules (which depend on Syk and ZAP-70). MHC-I engagement also inhibited, although more weakly, the activating signals triggered by the SAP-associated 2B4 activating receptor. Notably and unlike canonical inhibitory receptors, MHC-I has no inhibitory effect on the activating signals triggered by NKG2D (a DAP10-coupled specific activating receptor recruiting PI3K). In contrast to canonical inhibitory receptors, the MHC-I cytoplasmic tail is short and lacks consensus inhibitory signaling motifs. Nevertheless, it has been largely suggested that the aggregation of MHC-I proteins is able to induce positive and negative intracellular signals in T, B and NK cells [7]–[12], [21]–[28]. In our previous work, we detected that MHC-I crosslinking with anti-mouse IgG F(ab')_2_ on NKL cells induced tyrosine phosphorylation [10]. Moreover, the constitutive location of MHC-I proteins within lipid rafts on NKL cells [10], [29], as well as the MHC-I-specific inhibition of CD94-induced MTOC reorientation towards the P815:K14B06 contact area [11]–[12], strongly suggested that the inhibition of non-restricted cytotoxicity by aggregation of MHC class I molecules is mediated by intracellular inhibitory signals triggered by MHC-I. Consistent with these findings, it has recently been described that the constitutive expression of MHC class I molecules on murine macrophages inhibits the TLR4-triggered inflammatory response by association with the src kinase ftp and SHP-2 [13]. It has been reported that the cytoplasmic domain of MHC class I molecules is not needed for T cell signaling through these receptors, while the transmembrane region is indispensable for this effect [30]. Thus, it seems most likely that the MHC-I inhibitory function exerted upon ITAM-mediated NK cell cytotoxicity and IFN-γ secretion is mediated by either a lateral or *cis* association with some of the long list of cell surface molecules reported to physically associate with MHC-I proteins [31]–[32]. To date, it is unclear whether any type of MHC class I molecule is able to transduce inhibitory signals, or whether this property is limited to certain classical, non-classical, or even to their MHC-I open conformers bearing the monomorphic determinant recognized by anti-MHC-I mAb W6/32. MHC-I open conformers are unfolded molecules highly expressed on activated effector cells, where they form clusters through lateral or *cis* interaction with β2m-associated forms of MHC-I, as well as with non-classical HLA-F molecules, a feature that is likely to increase the avidity of any receptor recognition [33]–[35]. Moreover, open conformers are tyrosine phosphorylated probably mediated by Lck, since this src kinase is associated with HC-10 immunocomplexes [36]. Because KIR3DL2 and KIR2DS4 physically and functionally interact *in trans* with HLA-F and MHC-I open conformers [35], it is possible that these interactions also take place in *cis*, regulating KIR availability and activity. At this moment, we cannot totally exclude the involvement of open conformers in the inhibitory effect described here since we have not been able to obtain the specific mAbs. Nevertheless, our previous data from primary unstimulated human NK cells (in which the expression of open conformers is probably low) [10], together with the results obtained here with mAb that recognize β2m and those for the anti-HLAB27 mAb inhibition of CD94-redirected lysis of P815 by a CD8^+^αβ T cell clone [11]–[12] point to the involvement of classical trimeric human MHC-I molecules. Regarding *cis* interactions between MHC-I and inhibitory receptors, it has been reported from a murine model, that MHC-I molecules are recognized by Ly49 inhibitory receptors in *cis* and *trans* [32]. Furthermore, approximately 75% of the Ly49A receptors are masked by *cis* interaction with endogenous H-2D^d^ ligands [37] and, interestingly, the licensing of NK cells requires both *cis* and *trans* recognition of MHC class I molecules [38]. Although it is unclear whether this is a general feature in human NK cells, recent evidence has shown the *cis* association of LIR1/ILT2 with the MHC-I molecules that modulates the accessibility to antibodies and binding to the human CMV MHC-I homolog UL18 [39].

**Figure 5:**
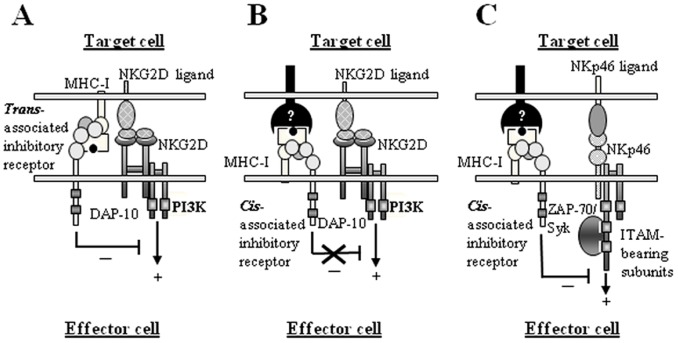
Model for MHC-I selective inhibition. (**A**) *Trans*-associated inhibitory receptors to MHC-I molecules are always inhibitory for effector cells. (**B**) and (**C**) *Cis*-associated inhibitory receptor/MHC-I selectively inhibits activating receptor signaling. It is proposed that LIRL receptors which bind to α3-β2m domains, and KIR or CD94-NKG2 receptors which bind to α1–α2 domains on MHC-I molecules [31] could participate in these interactions (shown as unknown receptors).

Our results suggest that a putative MHC-I/inhibitory receptor association in *cis* could dually regulate the activity of both inhibitory and activating receptors, in agreement with Held and Mariuzza [31]. Thus, constitutive MHC-I/Inhibitory receptor *cis* interactions could weaken inhibitory signals by reducing the ability of KIRs, LILRs and/or CD94/NKG2A to detect self ligands on target cells, as previously shown in murine NK cells [32], while selectively up-regulating their inhibitory capacity, as shown in **Figure 5C**. Our model proposes that inhibitory receptor-MHC-I interaction *in trans* would always be inhibitory for NK cells (**Fig. 5A**), whereas the same interaction *in cis* might be inhibitory, depending on the activating pathway triggered (**Fig. 5B** **** ***vs*** **5C**). The experimental approach used here could mimic a synaptic trimolecular complex that would be integrated by an effector MHC-I molecule associated *in cis* to a hypothetical LIRL or KIR receptor through the α3-β2m domains, and a KIR or CD94-NKG2 receptor (shown as unknown receptors) bound to the α1-α2 domains of the MHC-I molecule (reviewed in ref. [31]), as shown in **Fig. 5B and 5C**.

Related to these findings, we have recently identified that CD33 (either in *cis* or *trans*) acts as a unique fine-tune inhibitory receptor with the capacity to efficiently antagonize the cytotoxic response mediated by NKG2D (a DAP10-coupled specific activating receptor recruiting PI3K), or the SAP-associated 2B4 activating receptor, but not by CD16 or NKp46 receptors coupled to ITAM bearing subunits (depending on Syk and ZAP-70) (manuscript submitted). In addition, CD33 does not inhibit the IFN-γ production of NKL cells. Here, we show that, unlike but complementary to CD33, MHC-I inhibits both cytotoxicity and IFN-γ secretion on NK cells triggered by CD16, NKp46, and 2B4, but not by NKG2D. Consequently, we propose that CD33 and MHC-I belong to a new group of selective inhibitory receptors (**Figure 5B** *vs* **5C**), distinct from the best known canonical inhibitory receptors such as ILT2 or CD94/NKG2A, which efficiently regulate both the cytotoxicity and cytokine production triggered by all activating receptors, independently of the specific intermediates recruited.

Previously, we suggested that ILT2 (LILRB1, CD85j) and ILT4 (LILRB2, CD85d) proteins could be the principal MHC-I ligands candidates on APC to confer a suppressive effect on activated NK cells after ligation [10], [12]. It is possible that the resistance of mature DC to NK lysis could be related not only to the described up-regulation of MHC class I expression on their surface [40], but also to a hypothetically increased expression of LILRs.

In conclusion, this work describes for the first time a group of Killer cell selective inhibitory receptors in NK and activated T cells, which may be strongly involved in the regulation of immune responses against cancer and infected cells, in protecting self-cells and, probably, in avoiding autoimmunity. The selective nature of the inhibitory effect described provides new tools for dissecting the molecular mechanisms involved in cytotoxic cell inhibition. Further work is necessary to understand the integration of these multiple signals, the results of which will certainly improve our knowledge and ability to manipulate NK cell signaling pathways.

## Supporting Information

File S1 **Supporting figures. Figure S1, Crosslinking the NKL cell surface receptors CD58, CD54 (ICAM-1), CD50 (ICAM-3), CD29, CD44, CD2 and CD25 with the killer activating receptors, CD16, NKG2D and NKp46 did not significantly decrease the NKL cell-mediated cytotoxicity against P815 cells. Figure S2, MHC-I engagement augments NKL/P815 cell conjugation.** Exponentially growing Ca-AM-stained (calcein acetoxymethylester) NKL cells were co-cultured with HE-stained (hydroethidine) P815 cells plus mAb against Killer Activating Receptors or Inhibitory receptors at 1∶2 E/T ratio.
(DOCX)
