# Supplementary material for: MHC-I Molecules Selectively Inhibit Cell-Mediated Cytotoxicity Triggered by ITAM-Coupled Activating Receptors and 2B4
Source: PLoS One. 2014 Sep 16;9(9):e107054. doi: 10.1371/journal.pone.0107054 (PMC4166474; doi:10.1371/journal.pone.0107054)
Supplement: File S1 — Supporting figures. Figure S1, Crosslinking the NKL cell surface receptors CD58, CD54 (ICAM-1), CD50 (ICAM-3), CD29, CD44, CD2 and CD25 with the killer activating receptors, CD16, NKG2D and NKp46 did not significantly decrease the NKL cell-mediated cytotoxicity against P815 cells. Figure S2, MHC-I engagement augments NKL/P815 cell conjugation. Exponentially growing Ca-AM-stained (calcein acetoxymethylester) NKL cells were co-cultured with HE-stained (hydroethidine) P815 cells plus mAb against Killer Activating Receptors or Inhibitory receptors at 1∶2 E/T ratio. (DOCX) [file pone.0107054.s001.docx]

#### **Supporting Information**

**
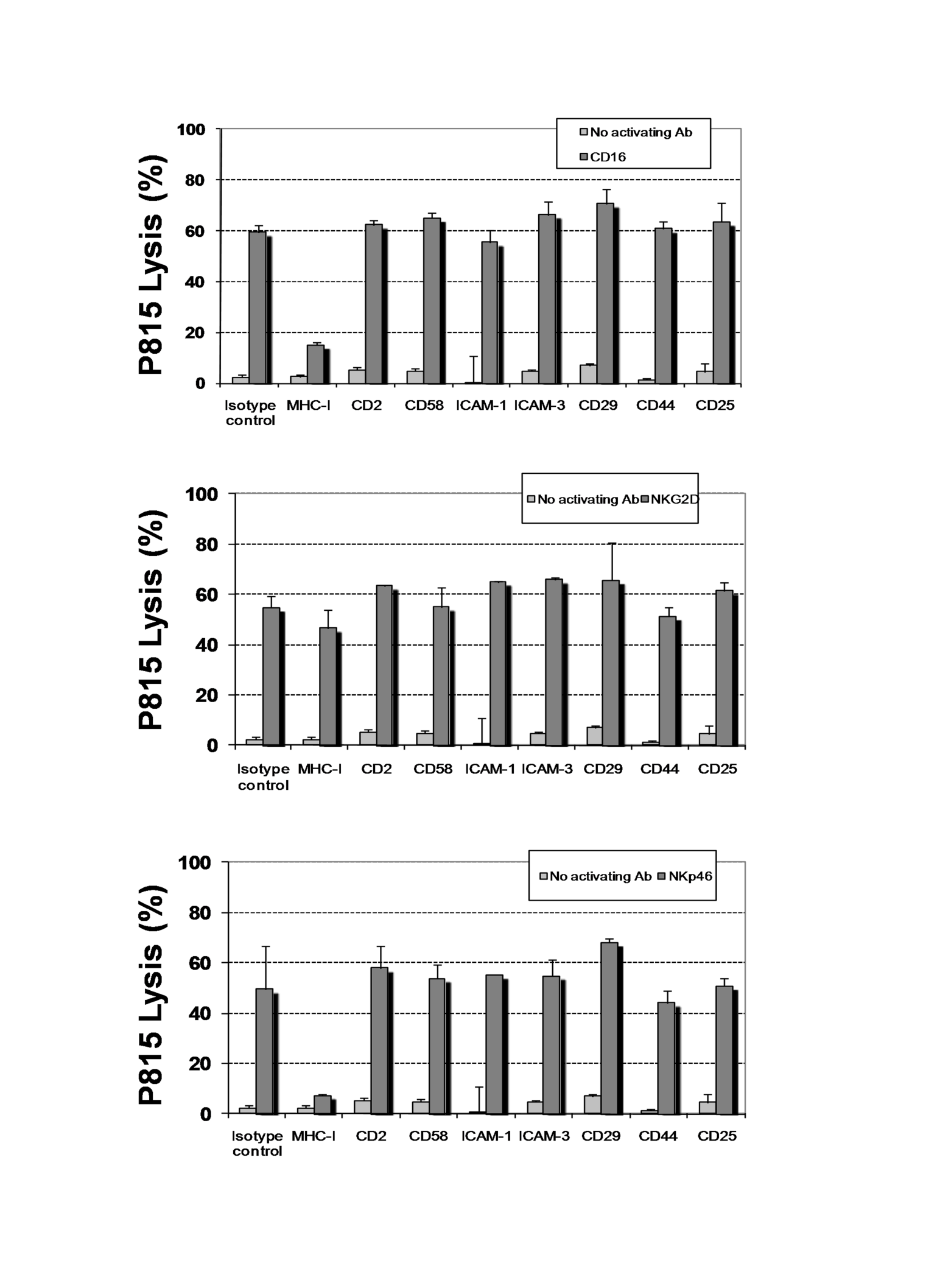
**

**FIGURE S1.-** Crosslinking the NKL cell surface receptors, CD58 (clone TS2/9), CD54 (ICAM-1) (clone R6.5), CD50 (ICAM-3) (clone HP2/19), CD29 (clone 4B4), CD44 (clone HP2/9), CD2 (clone TS2/18) and CD25 (clone MAR93), all of them at 1.25μg/ml final concentration, with the killer activating receptors, CD16, NKG2D and NKp46 did not significantly decrease the NKL cell-mediated cytotoxicity against P815 cells. NKL cells were co-cultured with ^51^Cr-P815 cells in the presence of mAb against Killer Activating Receptors (KAR): CD16, NKG2D and NKp46; plus isotype control IgG2a, MHC-I (W6/32 mAb), or the rest of mAb named above at 5:1 E/T ratio.

#### Effector/target conjugation assays

One day or two exponentially growing NKL cells were labeled green by incubation with 400 nM calcein acetoxymethylester (Ca-AM, Molecular Probes). P815 cells were labeled red with 235 μM hydroethidine (HE, Molecular Probes). After labeling, NKL cells were seeded with P815 cells on a 96-well round bottom plate at 1:2 E/T ratio in the absence or in the presence of the same combination of mAb, as used in the redirected cytotoxicity assays. Plated cells were centrifuged for 5 min at 500 rpm and incubated at room temperature for 20 min. Conjugation was stopped on ice and the percentage of conjugates was measured by flow cytometry. For each sample, a minimum of 8000 NKL cells was acquired. The percentage of bound cells (double positive events) was used to study E/T binding under the experimental conditions used in the NKL functional assays.

Spontaneous NKL/P815 conjugation (2 ± 0.1%) in the absence of mAb, and in the presence of Ab isotype control, anti-CD94 or anti-NKG2A mAb, was low or null, as shown in **Figure S2,** which is consistent with the low level of P815 killing obtained under these experimental conditions. As expected, the percentages of NKL/P815 conjugates increased in the presence of anti-MHC-I. Effector-target conjugation also increased in the presence of anti-NKG2D mAb, but was very weakly induced by anti-CD16, anti-NKp46 and anti-2B4 mAb (**Fig. S2**). Notably, co-engagement of anti-MHC-I with the activating receptors, CD16, NKG2D and 2B4, increased the percentages of conjugation in an additive form, indicating that under our experimental conditions there was no significant competition for P815 FcR. Furthermore, if this was the case, anti-MHC class I mAb would have strongly inhibited the NKG2D-triggered cytotoxicity, as is the most heavily expressed KAR on the membrane of NKL cells. Hence, these data show that the selective inhibition exerted by MHC-I molecules upon CD16-, NKp46- and 2B4-mediated NKL cytotoxicity in the reverse assays is not caused by a FcR competition phenomenon.

**
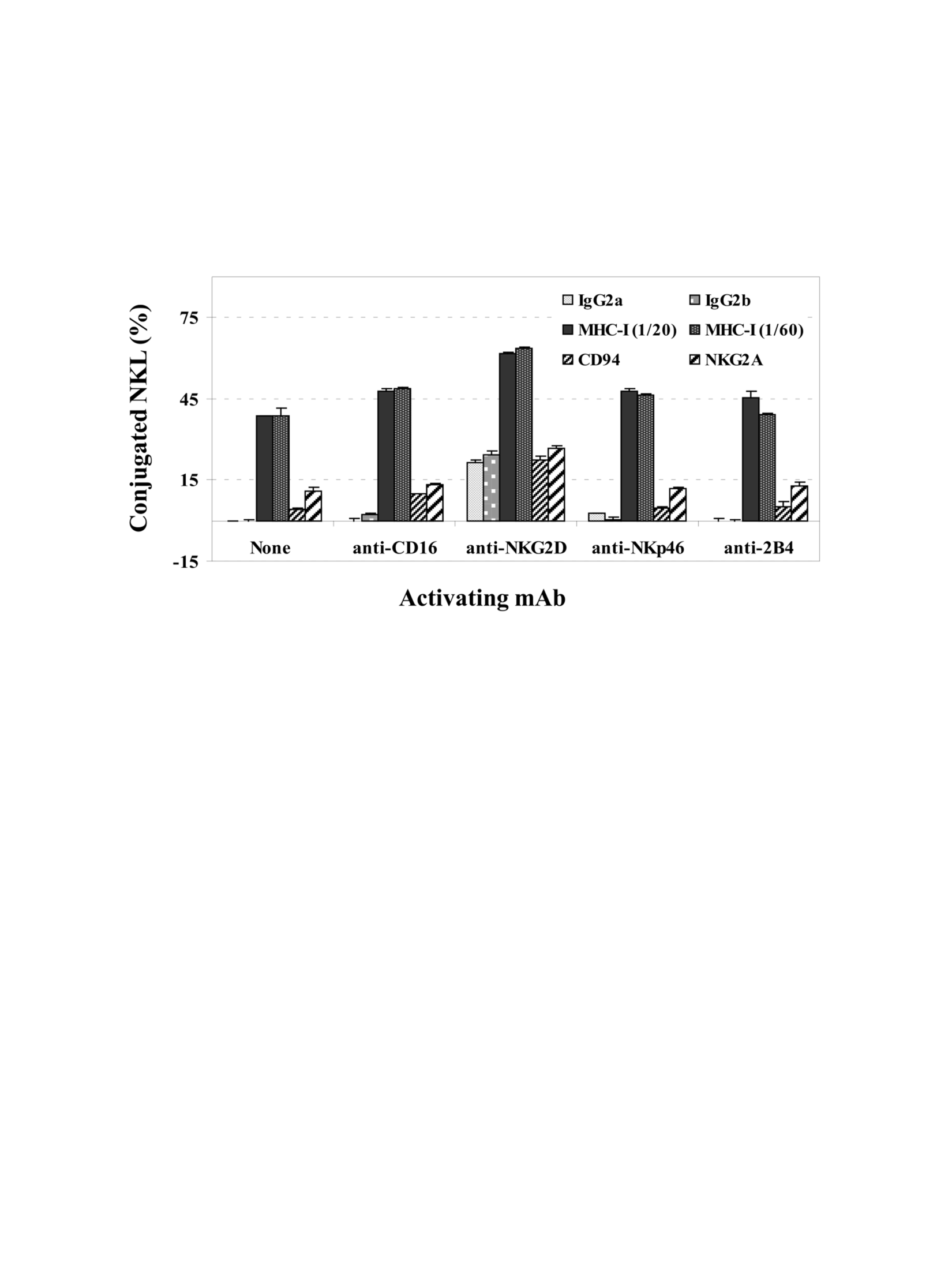
**

**FIGURE S2.- MHC-I engagement augments NKL/P815 cell conjugation.** Exponentially growing Ca-AM stained NKL cells were co-cultured with HE-stained P815 cells plus mAb against Killer Activating Receptors or Inhibitory receptors at 1:2 E/T ratio. The percentages of NKL cell conjugation (mean ± SD) are shown for each inhibitory receptor from one representative assay out of three performed with similar results. Numbers in parentheses are the supernatant dilution.
